# Crystal structure of *S*-(4-methyl­benz­yl) piperidine­dithio­carbamate

**DOI:** 10.1107/S2056989015014462

**Published:** 2015-08-06

**Authors:** Z. A. Rahima, Siti Nadiah Abdul Halim, Fiona N.-F. How

**Affiliations:** a Department of Chemistry, Kulliyyah of Science, International Islamic University Malaysia, 25200 Kuantan, Malaysia; b Department of Chemistry, Faculty of Science, Universiti Malaya, Kuala Lumpur 50603, Malaysia

**Keywords:** crystal structure, di­thio­carbamate, substituted di­thio­carbamate, piperidine di­thio­carbamate

## Abstract

The title compound, C_14_H_19_NS_2_, crystallizes in the thione form with the presence of a C=S bond. The piperidine ring adopts a chair conformation. The dihedral angle between the essentially planar di­thio­carbamate and *p*-tolyl fragments is 74.46 (10)°

## Related literature   

For the synthesis and related structures, see: Nabipour (2011 ▸); Kumar *et al.* (2013 ▸); Kotresh *et al.* (2012 ▸). For the various applications of di­thio­carbamates, see: Hogarth (2005 ▸).
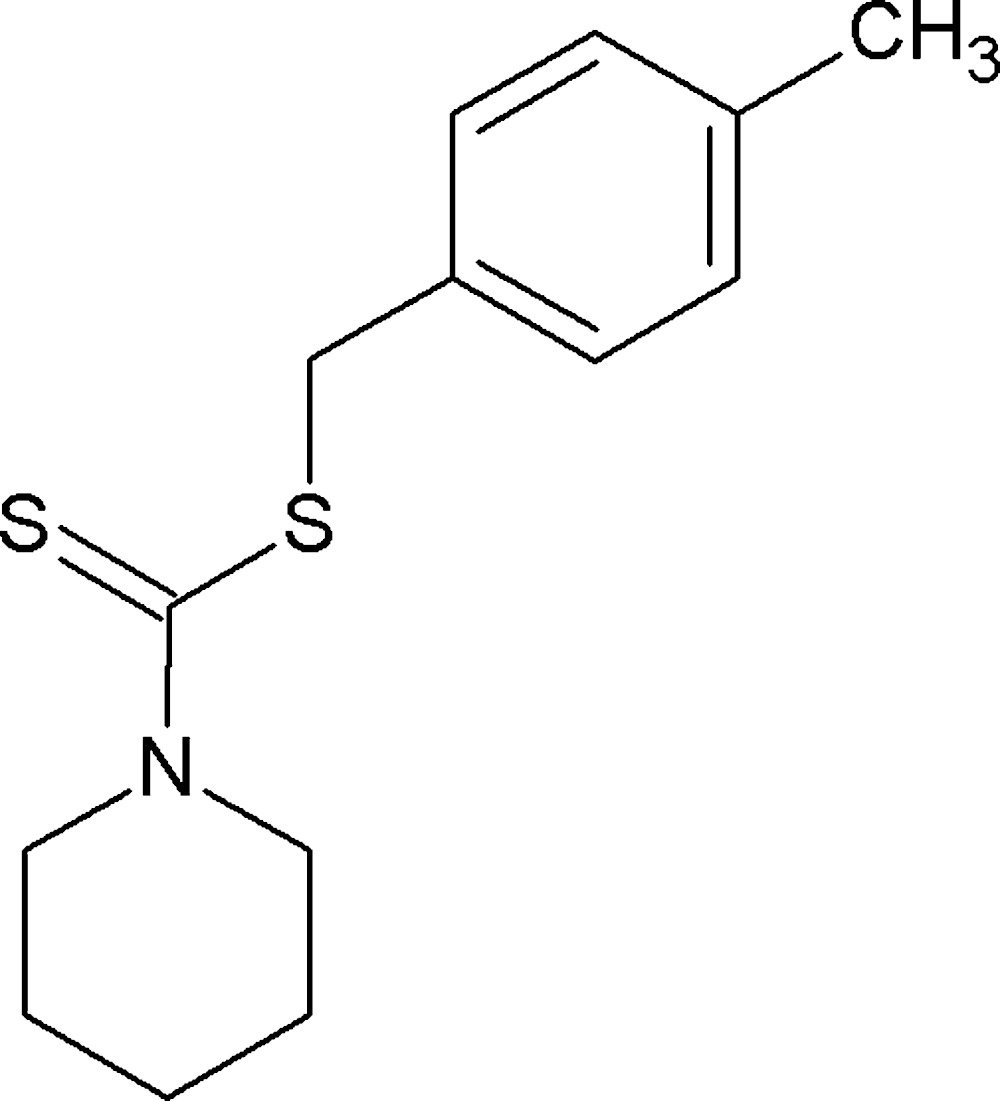


## Experimental   

### Crystal data  

C_14_H_19_NS_2_
*M* *_r_* = 265.42
Monoclinic, 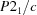

*a* = 6.3081 (4) Å
*b* = 11.2191 (7) Å
*c* = 19.8399 (13) Å
β = 96.133 (5)°
*V* = 1396.06 (15) Å^3^
*Z* = 4
Mo *K*α radiation
μ = 0.36 mm^−1^
*T* = 100 K
0.4 × 0.2 × 0.1 mm

### Data collection  

Bruker APEXII CCD diffractometer
Absorption correction: multi-scan (*SADABS*; Bruker, 2012 ▸) *T*_min_ = 0.666, *T*_max_ = 0.746
13322 measured reflections
3278 independent reflections
1866 reflections with *I* > 2σ(*I*)
*R*_int_ = 0.105

### Refinement  

*R*[*F* ^2^ > 2σ(*F* ^2^)] = 0.053
*wR*(*F* ^2^) = 0.114
*S* = 1.01
3278 reflections
155 parameters
H-atom parameters constrained
Δρ_max_ = 0.32 e Å^−3^
Δρ_min_ = −0.34 e Å^−3^

### 

Data collection: *APEX2* (Bruker, 2009 ▸); cell refinement: *SAINT* (Bruker, 2009 ▸); data reduction: *SAINT*; program(s) used to solve structure: *SIR2004* (Burla *et al.*, 2007 ▸); program(s) used to refine structure: *SHELXL97* (Sheldrick, 2008 ▸); molecular graphics: *Mercury* (Macrae *et al.*, 2008 ▸); software used to prepare material for publication: pubICIF (Westrip, 2010 ▸).

## Supplementary Material

Crystal structure: contains datablock(s) I. DOI: 10.1107/S2056989015014462/lh5777sup1.cif

Structure factors: contains datablock(s) I. DOI: 10.1107/S2056989015014462/lh5777Isup2.hkl

Supporting information file. DOI: 10.1107/S2056989015014462/lh5777Isup3.cml

**Fig. 1.**
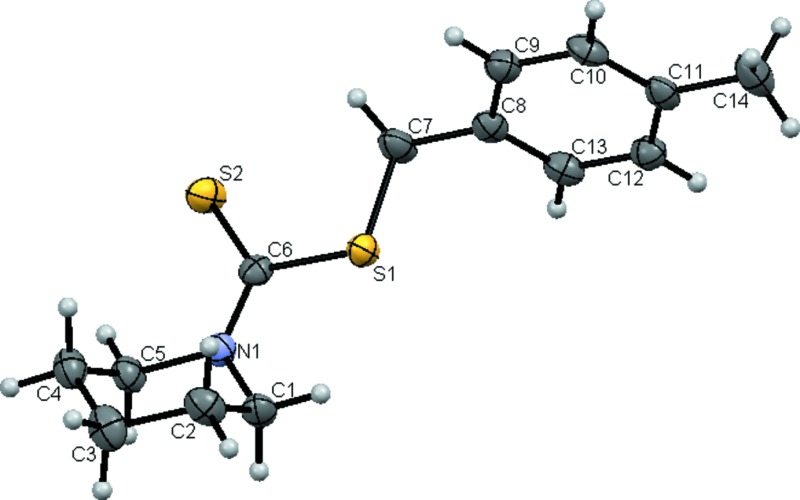
The molecular structure of the title compound with displacement ellipsoids drawn at the 50% probability level. H atoms are shown as spheres of arbitrary radius.

. DOI: 10.1107/S2056989015014462/lh5777fig1.tif
The mol­ecular structure of the title compound with displacement ellipsoids drawn at the 50% probability level. H atoms are shown as spheres of arbitrary radius.

CCDC reference: 975555

Additional supporting information:  crystallographic information; 3D view; checkCIF report

## supplementary crystallographic information

### S1. Comment

Di­thio­carbamates are well known to possess various properties with a wide range of applications (Hogarth, 2005). In our attempt to modify the substituents of piperidine di­thio­carbamate we have formed the title compound. It is likely that this compound is bioactive and will be an inter­est for further research.

The C6—S2 bond is 1.664 (3) Å, which is an inter­mediate of the standard value for C═S (1.56 Å) and shorter than a C—S single bond (1.82 Å). This is attributed to a slight delocalization of negative charge over the C—N—C—S chain.

The piperidine ring shows a chair conformation with Cremer-Pople puckering parameters Q= 0.583 (3) Å, θ= 2.9 (3)°, φ= 355 (6)°. The dihedral angle between the planar di­thio­carbamate moiety S1/S2/N1/C6 and the planar *p*-tolyl frgament C7/C8/C9/C10/C11/C12/C13/C14 is 74.46 (10)°. The C7–S1–C6–S2 fragment adopts a *cis* conformation with the torsion angle of -6.6 (2)° comparable to previous literature (Kumar *et al.*, 2013; Kotresh *et al.*, 2012). The arrangement of the molecules in the crystal are dominated by the presence of the crystallographic 2-fold rotation axis. There are no significant pi–pi inter­actions or other specific inter­molecular inter­actions in the crystal structure.

### S2. Experimental

Sodium piperidine di­thio­carbamate was pre-synthesized in accordance to the method of Nabipour (2011). Sodium piperidine di­thio­carbamate (5.5 mmol) in absolute ethanol (30 ml) was stirred continuously with dropwise addition of equimolar amounts of 4-methyl­benzyl chloride until a precipitation occurred. The precipitate (sodium chloride) was filtered off and the filtrate was reduced to half the volume and left to stand at room temperature to give colourless crystals. Yield= 63.11%, m.p.= 348.15–349.15 K. IR (KBr pellets, cm^–1^): 1477 (s, *ν* N–CSS), 1224 (s, *ν* C=S) and 978 (s, *ν* C–S). UV–Vis in CH_3_OH [λ_max_/nm, with ε (L mol^–1^ cm^–1^)]: 277 (2.05), 255 (4.09), 245 (4.20). ^1^H–NMR [DMSO–d_6_]: δ (ppm) = 4.46 (s, 2H, S–CH_2_–Bz); 4.22 (s, 2H, N–CH_2_), 3.85 (s, 2H, N–CH_2_); 7.11–7.27 (m, 4H, C_6_H_4_); 2.27 (s, 3H, CH_3_). ^13^C–NMR [DMSO–d_6_]: δ (ppm) = 193.82 (NCSS); 41.17 (S–CH_2_–Bz); 52.77, 51.33 (N–CH_2_); 129.49–137.03 (C aromatic); 21.18 (CH_3_).

### S2.1. Refinement

H-atoms were placed in calculated positions (C—H 0.93–0.97 Å) and were included in the refinement in a riding-model approximation with *U*_iso_(H) = 1.2*U*_eq_(C) or 1.2*U*_eq_(C_methyl_).

### Crystal data

**Table:**

C_14_H_19_NS_2_	*F*(000) = 568
*M**_r_* = 265.42	*D*_x_ = 1.263 Mg m^−^^3^
Monoclinic, *P*2_1_/*c*	Mo *K*α radiation, λ = 0.71073 Å
*a* = 6.3081 (4) Å	Cell parameters from 883 reflections
*b* = 11.2191 (7) Å	θ = 3.6–20.8°
*c* = 19.8399 (13) Å	µ = 0.36 mm^−^^1^
β = 96.133 (5)°	*T* = 100 K
*V* = 1396.06 (15) Å^3^	Block, colourless
*Z* = 4	0.4 × 0.2 × 0.1 mm

### Data collection

**Table:**

Bruker APEXII CCD diffractometer	3278 independent reflections
Radiation source: sealed tube	1866 reflections with *I* > 2σ(*I*)
Graphite monochromator	*R*_int_ = 0.105
Detector resolution: 8 pixels mm^-1^	θ_max_ = 27.9°, θ_min_ = 2.1°
φ and ω scans	*h* = −8→8
Absorption correction: multi-scan *SADABS* (Bruker, 2012)	*k* = −14→14
*T*_min_ = 0.666, *T*_max_ = 0.746	*l* = −25→25
13322 measured reflections	

### Refinement

**Table:**

Refinement on *F*^2^	Primary atom site location: iterative
Least-squares matrix: full	Secondary atom site location: difference Fourier map
*R*[*F*^2^ > 2σ(*F*^2^)] = 0.053	Hydrogen site location: inferred from neighbouring sites
*wR*(*F*^2^) = 0.114	H-atom parameters constrained
*S* = 1.01	*w* = 1/[σ^2^(*F*_o_^2^) + (0.036*P*)^2^ + 0.1195*P*] where *P* = (*F*_o_^2^ + 2*F*_c_^2^)/3
3278 reflections	(Δ/σ)_max_ < 0.001
155 parameters	Δρ_max_ = 0.32 e Å^−^^3^
0 restraints	Δρ_min_ = −0.34 e Å^−^^3^

### Special details

**Table:**

Geometry. All e.s.d.'s (except the e.s.d. in the dihedral angle between two l.s. planes) are estimated using the full covariance matrix. The cell e.s.d.'s are taken into account individually in the estimation of e.s.d.'s in distances, angles and torsion angles; correlations between e.s.d.'s in cell parameters are only used when they are defined by crystal symmetry. An approximate (isotropic) treatment of cell e.s.d.'s is used for estimating e.s.d.'s involving l.s. planes.
Refinement. Refinement of *F*^2^ against ALL reflections. The weighted *R*-factor *wR* and goodness of fit *S* are based on *F*^2^, conventional *R*-factors *R* are based on *F*, with *F* set to zero for negative *F*^2^. The threshold expression of *F*^2^ > σ(*F*^2^) is used only for calculating *R*-factors(gt) *etc*. and is not relevant to the choice of reflections for refinement. *R*-factors based on *F*^2^ are statistically about twice as large as those based on *F*, and *R*-factors based on ALL data will be even larger.

### Fractional atomic coordinates and isotropic or equivalent isotropic displacement parameters (Å^2^)

**Table:**

	*x*	*y*	*z*	*U*_iso_*/*U*_eq_	
S1	0.60407 (11)	0.46509 (6)	0.32887 (4)	0.0264 (2)	
S2	0.40927 (14)	0.70252 (7)	0.35563 (4)	0.0347 (2)	
N1	0.3416 (3)	0.5804 (2)	0.24001 (12)	0.0255 (6)	
C7	0.7321 (5)	0.5087 (3)	0.41139 (15)	0.0339 (8)	
H7A	0.8107	0.5824	0.4080	0.041*	
H7B	0.6267	0.5204	0.4430	0.041*	
C13	1.0788 (5)	0.3979 (3)	0.41109 (14)	0.0297 (7)	
H13	1.1215	0.4545	0.3811	0.036*	
C8	0.8810 (5)	0.4086 (2)	0.43476 (14)	0.0266 (7)	
C6	0.4390 (4)	0.5890 (2)	0.30345 (14)	0.0240 (7)	
C12	1.2139 (5)	0.3046 (3)	0.43127 (14)	0.0298 (7)	
H12	1.3454	0.2991	0.4143	0.036*	
C3	−0.0478 (5)	0.4953 (3)	0.16795 (17)	0.0382 (8)	
H3A	−0.1839	0.4561	0.1702	0.046*	
H3B	−0.0354	0.5148	0.1209	0.046*	
C9	0.8232 (5)	0.3239 (3)	0.48066 (15)	0.0305 (7)	
H9	0.6921	0.3301	0.4979	0.037*	
C14	1.3055 (5)	0.1182 (3)	0.49932 (17)	0.0429 (9)	
H14A	1.3890	0.0976	0.4633	0.064*	
H14B	1.2243	0.0502	0.5108	0.064*	
H14C	1.3983	0.1429	0.5383	0.064*	
C5	0.1796 (4)	0.6661 (3)	0.21235 (15)	0.0299 (7)	
H5A	0.2074	0.6898	0.1671	0.036*	
H5B	0.1853	0.7368	0.2407	0.036*	
C1	0.3454 (5)	0.4735 (3)	0.19726 (15)	0.0305 (7)	
H1A	0.4574	0.4201	0.2160	0.037*	
H1B	0.3747	0.4960	0.1520	0.037*	
C2	0.1319 (5)	0.4110 (3)	0.19406 (16)	0.0345 (8)	
H2A	0.1086	0.3829	0.2389	0.041*	
H2B	0.1320	0.3425	0.1643	0.041*	
C11	1.1563 (5)	0.2185 (3)	0.47668 (14)	0.0283 (7)	
C10	0.9587 (5)	0.2306 (3)	0.50101 (14)	0.0307 (7)	
H10	0.9166	0.1748	0.5316	0.037*	
C4	−0.0398 (5)	0.6094 (3)	0.20983 (16)	0.0344 (8)	
H4A	−0.1466	0.6648	0.1899	0.041*	
H4B	−0.0716	0.5913	0.2555	0.041*	

### Atomic displacement parameters (Å^2^)

**Table:**

	*U*^11^	*U*^22^	*U*^33^	*U*^12^	*U*^13^	*U*^23^
S1	0.0309 (4)	0.0220 (4)	0.0259 (4)	0.0032 (3)	0.0012 (3)	−0.0015 (3)
S2	0.0515 (5)	0.0223 (4)	0.0299 (5)	0.0053 (4)	0.0030 (4)	−0.0025 (3)
N1	0.0258 (13)	0.0230 (13)	0.0272 (14)	0.0022 (11)	0.0009 (11)	−0.0022 (11)
C7	0.0435 (19)	0.0309 (18)	0.0250 (17)	0.0050 (15)	−0.0067 (14)	−0.0050 (14)
C13	0.0354 (18)	0.0271 (17)	0.0255 (17)	−0.0063 (14)	−0.0015 (14)	0.0020 (13)
C8	0.0323 (17)	0.0218 (16)	0.0247 (16)	−0.0006 (14)	−0.0019 (13)	−0.0047 (13)
C6	0.0267 (16)	0.0218 (15)	0.0243 (16)	−0.0014 (13)	0.0066 (13)	0.0020 (12)
C12	0.0280 (16)	0.0341 (18)	0.0271 (17)	−0.0025 (15)	0.0023 (13)	−0.0009 (14)
C3	0.0307 (17)	0.0354 (19)	0.047 (2)	−0.0068 (14)	−0.0010 (15)	0.0049 (16)
C9	0.0315 (17)	0.0338 (18)	0.0260 (17)	−0.0003 (15)	0.0016 (13)	−0.0018 (14)
C14	0.048 (2)	0.0357 (19)	0.041 (2)	0.0072 (17)	−0.0157 (17)	−0.0013 (16)
C5	0.0329 (17)	0.0254 (16)	0.0306 (18)	0.0045 (14)	−0.0009 (14)	0.0040 (14)
C1	0.0332 (17)	0.0306 (17)	0.0264 (17)	0.0035 (15)	−0.0022 (13)	−0.0069 (14)
C2	0.0395 (19)	0.0282 (17)	0.0341 (19)	−0.0037 (15)	−0.0041 (15)	−0.0013 (14)
C11	0.0303 (17)	0.0277 (16)	0.0247 (17)	−0.0011 (14)	−0.0073 (13)	−0.0037 (13)
C10	0.0408 (19)	0.0262 (17)	0.0240 (17)	−0.0052 (15)	−0.0017 (14)	0.0056 (13)
C4	0.0304 (17)	0.0333 (18)	0.039 (2)	0.0040 (15)	0.0025 (14)	0.0084 (15)

### Geometric parameters (Å, º)

**Table:**

S1—C7	1.814 (3)	C9—H9	0.9300
S1—C6	1.778 (3)	C9—C10	1.384 (4)
S2—C6	1.664 (3)	C14—H14A	0.9600
N1—C6	1.344 (3)	C14—H14B	0.9600
N1—C5	1.466 (3)	C14—H14C	0.9600
N1—C1	1.471 (3)	C14—C11	1.504 (4)
C7—H7A	0.9700	C5—H5A	0.9700
C7—H7B	0.9700	C5—H5B	0.9700
C7—C8	1.505 (4)	C5—C4	1.519 (4)
C13—H13	0.9300	C1—H1A	0.9700
C13—C8	1.384 (4)	C1—H1B	0.9700
C13—C12	1.382 (4)	C1—C2	1.513 (4)
C8—C9	1.392 (4)	C2—H2A	0.9700
C12—H12	0.9300	C2—H2B	0.9700
C12—C11	1.395 (4)	C11—C10	1.391 (4)
C3—H3A	0.9700	C10—H10	0.9300
C3—H3B	0.9700	C4—H4A	0.9700
C3—C2	1.523 (4)	C4—H4B	0.9700
C3—C4	1.524 (4)		
			
C6—S1—C7	103.63 (13)	C11—C14—H14A	109.5
C6—N1—C5	122.3 (2)	C11—C14—H14B	109.5
C6—N1—C1	124.4 (2)	C11—C14—H14C	109.5
C5—N1—C1	111.9 (2)	N1—C5—H5A	109.8
S1—C7—H7A	110.5	N1—C5—H5B	109.8
S1—C7—H7B	110.5	N1—C5—C4	109.5 (2)
H7A—C7—H7B	108.7	H5A—C5—H5B	108.2
C8—C7—S1	106.24 (19)	C4—C5—H5A	109.8
C8—C7—H7A	110.5	C4—C5—H5B	109.8
C8—C7—H7B	110.5	N1—C1—H1A	109.8
C8—C13—H13	119.4	N1—C1—H1B	109.8
C12—C13—H13	119.4	N1—C1—C2	109.4 (2)
C12—C13—C8	121.2 (3)	H1A—C1—H1B	108.2
C13—C8—C7	121.1 (3)	C2—C1—H1A	109.8
C13—C8—C9	118.1 (3)	C2—C1—H1B	109.8
C9—C8—C7	120.8 (3)	C3—C2—H2A	109.5
S2—C6—S1	121.57 (17)	C3—C2—H2B	109.5
N1—C6—S1	113.9 (2)	C1—C2—C3	110.7 (2)
N1—C6—S2	124.6 (2)	C1—C2—H2A	109.5
C13—C12—H12	119.4	C1—C2—H2B	109.5
C13—C12—C11	121.1 (3)	H2A—C2—H2B	108.1
C11—C12—H12	119.4	C12—C11—C14	120.9 (3)
H3A—C3—H3B	108.1	C10—C11—C12	117.5 (3)
C2—C3—H3A	109.5	C10—C11—C14	121.6 (3)
C2—C3—H3B	109.5	C9—C10—C11	121.4 (3)
C2—C3—C4	110.8 (3)	C9—C10—H10	119.3
C4—C3—H3A	109.5	C11—C10—H10	119.3
C4—C3—H3B	109.5	C3—C4—H4A	109.6
C8—C9—H9	119.6	C3—C4—H4B	109.6
C10—C9—C8	120.7 (3)	C5—C4—C3	110.3 (3)
C10—C9—H9	119.6	C5—C4—H4A	109.6
H14A—C14—H14B	109.5	C5—C4—H4B	109.6
H14A—C14—H14C	109.5	H4A—C4—H4B	108.1
H14B—C14—H14C	109.5		
			
S1—C7—C8—C13	79.9 (3)	C6—N1—C1—C2	104.9 (3)
S1—C7—C8—C9	−99.8 (3)	C12—C13—C8—C7	−178.5 (3)
N1—C5—C4—C3	−57.0 (3)	C12—C13—C8—C9	1.3 (4)
N1—C1—C2—C3	56.7 (3)	C12—C11—C10—C9	0.5 (4)
C7—S1—C6—S2	−6.6 (2)	C14—C11—C10—C9	179.1 (3)
C7—S1—C6—N1	174.4 (2)	C5—N1—C6—S1	172.6 (2)
C7—C8—C9—C10	178.7 (3)	C5—N1—C6—S2	−6.4 (4)
C13—C8—C9—C10	−1.1 (4)	C5—N1—C1—C2	−61.5 (3)
C13—C12—C11—C14	−179.0 (3)	C1—N1—C6—S1	7.5 (3)
C13—C12—C11—C10	−0.3 (4)	C1—N1—C6—S2	−171.5 (2)
C8—C13—C12—C11	−0.6 (4)	C1—N1—C5—C4	61.8 (3)
C8—C9—C10—C11	0.3 (4)	C2—C3—C4—C5	53.9 (3)
C6—S1—C7—C8	−178.0 (2)	C4—C3—C2—C1	−53.9 (3)
C6—N1—C5—C4	−105.1 (3)		
