# Supplementary figures and images for: Crystal structure of S-(4-methyl­benz­yl) piperidine­dithio­carbamate
Source: Acta Crystallogr E Crystallogr Commun. 2015 Aug 6;71(Pt 9):o647. doi: 10.1107/S2056989015014462 (PMC4555435; doi:10.1107/S2056989015014462)

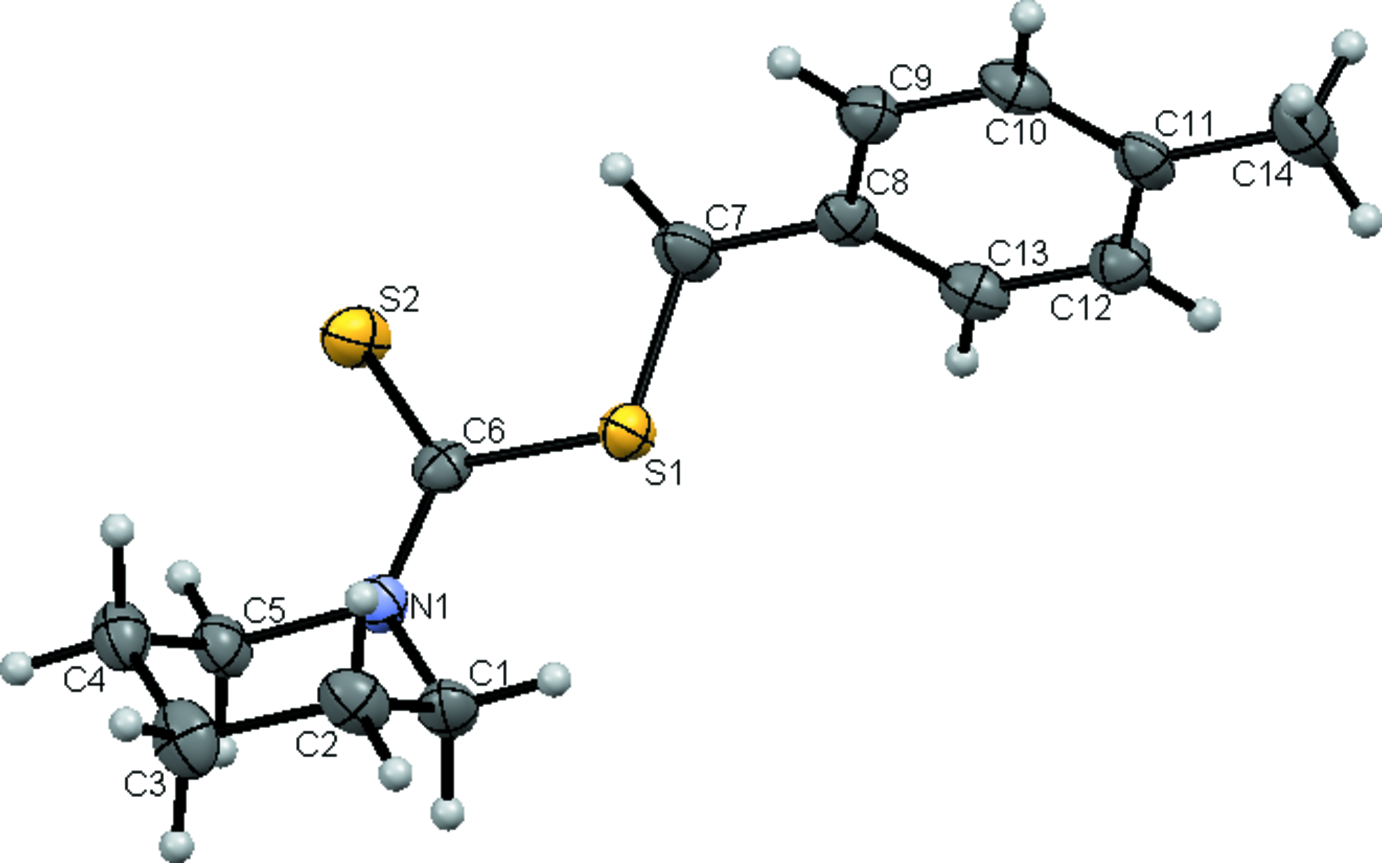

Supplement: Supplementary file 4 [file e-71-0o647-fig1.tif]
